# A Qualitative Evaluation of Caregivers’ Perceptions of a New Australian Public Mental Health Service (Nurturing Connections)

**DOI:** 10.3390/ijerph23070930

**Published:** 2026-07-20

**Authors:** Sara Cibralic, Phone Myat, Lulu Barker, Tracey Fay-Stammbach, Angeline Landry, Lee Meredith, Danielle Pretty, Ashleigh Allan, Jacinta Heath, Valsamma Eapen

**Affiliations:** 1 Psychiatry and Mental Health, School of Clinical Medicine, University of New South Wales, Sydney, NSW 2052, Australiav.eapen@unsw.edu.au (V.E.); 2 Academic Unit of Child Psychiatry (AUCS), South Western Sydney Local Health District, Liverpool, NSW 2170, Australia; 3 Ingham Institute for Applied Medical Research, Liverpool, NSW 2170, Australia; 4 Perinatal & Infant Mental Health, Mental Health Branch, New South Wales Ministry of Health, Sydney, NSW 2065, Australia; 5 Nurturing Connections, Mid North Coast Local Health District, Kempsey, NSW 2440, Australia; 6 Nurturing Connections, Northern Sydney Local Health District, Macquarie Hospital, North Ryde, NSW 2113, Australia; 7 Nurturing Connections, South Eastern Sydney Local Health District, Hurstville Community Health Care, Hurstville, NSW 2220, Australia

**Keywords:** perinatal mental health, infant health and development, multidisciplinary intervention, child–caregiver relationship, program development

## Abstract

**Highlights:**

**Public health relevance—how does this work relate to a public health issue?**

- Mental illness, particularly during early childhood, can have adverse long-term impacts on caregivers, their children, and their partners.
- The Nurturing Connections Program was developed to fill a service-needs gap for caregivers with mental health challenges, complex needs, and young children.

**Public health significance—why is this work of significance to public health?**

- Improving caregiver mental health during early parenthood can improve caregiver outcomes and have positive flow-on effects for the caregiving environment.
- Positive changes in the caregiving environment can lead to improvements in child health and development.

**Public health implications—what are the key implications or messages for practitioners, policy makers and/or researchers in public health?**

- Caregivers noted improvements in their mental health, their child’s development, and their interpersonal relationships following their engagement with the Nurturing Connections Program.
- The Nurturing Connections Program was found to be an acceptable and beneficial service.

**Abstract:**

Children whose primary caregivers experience mental illness and adverse psychosocial complexity are at increased risk of experiencing mental, physical, and relational challenges. Perinatal mental health services have commonly focused on treating the parental mental health problem; however, emerging evidence shows that parental mental health interventions alone are not sufficient to address the complex needs of both caregivers and their children. To address this gap in services, a new, highly specialised program—the Nurturing Connections Program—was implemented across three Australian local health districts within the State of New South Wales. The program is targeted to caregivers of young children referred to mental health services and aims to improve caregiver mental health and psychosocial adversity, child developmental outcomes, and the child–caregiver relationship. This study evaluated caregivers’ experiences with, and views of, the program. Twelve caregivers participated in semi-structured, in-depth interviews. A reflexive thematic analysis approach guided data interpretation. Four themes and three subthemes emerged from the data: (1) motivation for attending the program; (2) program components received; (3) post-program outcomes (subthemes: Caregiver-related outcomes, Child-related outcomes, and interpersonal outcomes); and (4) areas for program improvement. This study’s findings highlight program benefits, program facilitators and areas for improvement.

## 1. Introduction

The perinatal and early childhood years pose a heightened risk of the emergence or re-emergence of mental health illnesses among women [1,2]. It is well recognised that perinatal mental illness can have adverse consequences for primary caregivers, their children, and their partners [3,4]. In particular, some perinatal mental illnesses have been found to negatively impact the bond between caregivers and their children (often referred to as the caregiver–child attachment relationship) [5,6]. Children from families with caregivers experiencing mental health conditions and additional complex needs are at a greater risk of developing insecure and/or disorganised attachment relationships (linked to inconsistent and frightened/frightening caregiving) and the lifelong associated negative impacts [5,6]. Research is increasingly finding that addressing only the caregiver’s mental illness does not lead to improvements in the caregiver–child attachment relationship or child development [7,8]. Consequently, there is a need for perinatal mental health interventions that prioritise the relationship between caregivers and their children, target child development, and address the known social determinants of health.

In Australia, caregivers who are in an acute phase of a mental illness during the antenatal and postnatal periods can access treatment from Perinatal and Infant Mental Health (PIMH) Services [9]. PIMH services are public health services that support caregivers from preconception until their child is 12–24 months old, delivering interventions aimed at treating caregiver mental health, improving the caregiver–child attachment relationship, and supporting healthy child development [10,11]. Research has found PIMH services to be effective at improving both caregiver and child outcomes, including improving caregiver mental illness, increasing caregiver knowledge and parenting confidence, and improving the child–caregiver bond [12,13]. However, families who do not meet acuity criteria (i.e., are not experiencing the acute phase of a mental illness during the antenatal/postnatal period) or have older children (>24 months) are often ineligible to access this service. While children over the age of 2 years are able to access Child and Adolescent Mental Health Services (CAMHS) with their caregivers, CAMHS clinicians often lack the training in relational approaches (e.g., attachment-focused interventions involving dyads) needed to work with children under 5 years of age [9]. It has also been recognised that caregivers with a history of moderate-to-severe mental health illness and high psychosocial vulnerability require a longer duration of care and additional wrap-around support, including access to social care services [14]. The lack of long-term, comprehensive care has been a long-standing criticism of PIMH services [15]. Another criticism has been the lack of service provider capacity to provide programs suitable for Aboriginal and Torres Strait Islander families and families from culturally and linguistically diverse backgrounds [15], populations that are at an increased risk of experiencing mental health challenges during the perinatal period [16].

In recent years, governments have made investments aimed at improving access and expanding interventions offered to caregivers with moderate-to-severe/complex mental health difficulties, additional vulnerabilities, and young children [9]. Examples implemented in Australia in recent years include the Together in Mind intervention [17], implemented by the Queensland Centre for Perinatal and Infant Mental Health, and the Infant Access Program, developed and delivered by CAMHS in Victoria [9]. Both programs target at-risk families, with the Infant Access Program facilitating access to specialist mental health services and Together in Mind providing a 6-week intensive intervention addressing caregiver mental health, caregiving practices, and infant mental health. Furthermore, both programs have been integrated into existing services, which has been shown to improve accessibility and acceptability for at-risk populations [9]. Neither service, however, provides the long-term care recommended for high-risk populations.

### The Nurturing Connections Program

Nurturing Connections is a new program that was developed and launched in 2024 in the New South Wales (NSW) state of Australia [18]. It is a highly specialised public mental health service that provides long-term treatment and support to caregivers who have young children (pregnancy to school age), are experiencing moderate-to-severe mental health challenges, and have complex psychosocial needs, often in conjunction with or as a result of past and/or current trauma experiences. Following a competitive Expression of Interest process, the state-funded program was implemented across two metropolitan and one rural local health district (LHD)—South Eastern Sydney, Northern Sydney, and the Mid North Coast, respectively.

The Nurturing Connections Program was designed to bridge a service-needs gap in the existing statewide PIMH services [12,19]. To do so, the program expanded upon the existing PIMH service model by including broader referral criteria, longer program durations (6–9 months versus 3 months), and integrating social support. Furthermore, unlike existing services which are led by psychiatrists and comprised of mental health clinicians and case workers, the Nurturing Connections teams are led by senior mental health clinicians and staffed by multidisciplinary teams, including a child and family health nurse, perinatal and infant mental health clinicians, social workers, perinatal peer support workers, bilingual and multicultural mental health clinicians and/or Aboriginal mental health clinicians [20]. The program is delivered in partnership with non-government organisations which provide social and welfare support to address clients’ psychosocial needs/social determinants of health.

In-line with biopsychosocial–cultural, trauma-informed and attachment models, the program recognises that caregiver and child outcomes are impacted by numerous factors. A variety of interventions are, therefore, available to support caregiver mental health, the caregiver–child attachment relationship, and child development. These include attachment-focused therapies (i.e., Building Early Attachments and Resilience Support [21], Happiness, Understanding, Giving, and Sharing [22], and Parents Under Pressure [23]), perinatal peer support, psychiatric support, and child developmental screening. Interventions are provided based on a tiered/stepped model of care [24], whereby services are graded from low-intensity (e.g., groups) to high-intensity (e.g., specialised interventions) and provided on a needs basis as determined by a combination of standardised measures and clinical judgment. Strategies to enhance engagement are also employed, such as home visiting and arranging transport.

Given that the Nurturing Connections Program is a new program, caregiver perspectives on the program have not yet been evaluated. Understanding caregivers’ views is critical to understanding the program’s acceptability and feasibility, as well as facilitators and barriers to service access [25,26]. Drawing on a qualitative methodological approach, this study aimed to understand caregivers’ experience with and views of the Nurturing Connections Program during the early implementation phase. This study forms part of the broader mixed-methods evaluation of the Nurturing Connections Program [27].

## 2. Method

### 2.1. Setting

The South Eastern Sydney LHD (covering an area of 468 km^2^) [28] and Northern Sydney LHD (covering an area of 900 km^2^) [29] are urban areas that cater to populations of 979,370 and 985,708 people, respectively. The South Eastern Sydney Nurturing Connections team is located in Hurstville, while the Northern Sydney team is located within the Macquarie Hospital campus. Conversely, the Mid North Coast LHD is a rural health district (covering an area of 11,335 km^2^) which caters to a population of 226,422 people, with 7.5% being from Aboriginal and/or Torres Strait Islander backgrounds [30]. The services are managed by a full-time senior perinatal mental health clinician and staffed by a multidisciplinary team. Please see the published protocol paper for a detailed description of the service setting [27].

In the first year of operation (October 2023–October 2024), across the three LHDs, 241 caregivers were referred, and 196 met service eligibility criteria (see Table 1 for eligibility criteria) and were accepted into the program. This included 17 fathers, 15 women who were pregnant, and four kinship carers. Most referred caregivers had experienced complex early trauma, including physical, sexual, and/or emotional abuse and neglect, and/or systemic trauma through negative experiences with child protection, legal, health, and/or education services. In addition to experiencing early life trauma, caregivers presented with a range of mental health diagnoses, including anxiety, depression, adjustment, bipolar, obsessive–compulsive, schizophrenia, substance use, and neurodevelopmental disorders. Caregivers also had multiple psychosocial needs, such as family and domestic violence; unstable housing and homelessness; financial stress; isolation; being from a culturally and linguistically diverse and/or refugee background; having a history with the criminal justice system; being a single parent; and/or caring for a child with an illness or disability.

### 2.2. Participants

Twelve caregivers who had engaged with the Nurturing Connections Program consented to taking part in a qualitative interview aimed at exploring their experiences and perceptions of the program. All participants identified as female: 11 were biological mothers of the presenting child, and one was a maternal grandmother. Most participants had one (*n* = 8, ~66%) or two (*n* = 3, 25%) children. One participant (~8%) had one grandchild (presenting child). The caregivers ranged in age from 27 to 65 years (35.17 years, SD = 10.44), and the children were aged between 4 and 54 months (M age = 20.07 months, SD = 13.80). Three participants were Australian, two were from England, one was from the United States of America, one was from Georgia, one was from Lebanon, one was from Sierra Leone, one was from Panama, one was from Yugoslavia, and one identified as a Torres Strait Islander.

### 2.3. Procedure

Ethical Approval. This study formed part of a wider evaluation of the Nurturing Connections Program [27]. Ethical approval was obtained from the South Eastern Sydney Local Health District Research Ethics Committee (2024/ETH01715). Aboriginal Health and Medical Research Council of New South Wales (2380/25) approval was also received for the Mid North Coast site, which has a high percentage of Aboriginal and Torres Strait Islander service users.

Participant Recruitment. Purposive sampling was used to recruit caregivers who had taken part in the Nurturing Connections Program. This included caregivers who had completed the program or disengaged from the service early. The caregivers were informed about the qualitative component of the evaluation by their treating clinicians in person during their last treatment session or via telephone during a follow-up call subsequent to treatment disengagement. Clinicians made an effort to inform all service users of the evaluation. However, given the workload faced by treating clinicians in public mental health services, it was not within the clinician’s capacity to monitor the number of caregivers informed about the study or why caregivers declined to be contacted by the research team. Details of caregivers who expressed interest in participation were forwarded to the project lead (first author, SC), who telephoned the caregivers to provide further information about the current study and broader evaluation. It was also explained to caregivers that their decision regarding whether to participate in the research would not impact their relationship with the Nurturing Connections service. For caregivers who agreed to participate, an interview appointment was then agreed upon at a date and time that was suitable for them. A total of 16 participants were contacted: 12 agreed to participate, 3 were unable to be reached, and 1 reengaged with the service.

Semi-structured interviews. The interviews were conducted via telephone by a clinical psychologist and academic researcher with lived experience of parenting young children (SC). A semi-structured interview approach was used to explore the participants’ experiences with and perceptions of the service. This approach allowed for common topics to be covered across participants and the investigation of areas of interest, but also provided flexibility to explore novel topics that emerged. The interview questions were developed by SC based on the broader evaluation aims and implementation evaluation metrics as outlined by Proctor et al. [32]. The questions were then refined following feedback from the research team. The questions were focused on (1) reasons for attending the service (“Why did you decide to attend the Nurturing Connections Service?”), (2) experiences with the service (“What was your experience of the Nurturing Connections service?”), and (3) ways to improve the service (“Is there anything you think could be improved?”). Interviews took place between September and December 2025 and ranged in length from approximately 10 to 32 min (average length = ~18 min). Data was collected until saturation was reached [33]. The participants provided verbal, informed consent prior to taking part in the interview and received a $30 gift voucher for participation. The interviews were recorded and transcribed verbatim using Zoom’s (Zoom Video Communications, 2024; version 7.1.0).) inbuilt transcription software. All transcripts were then manually reviewed by PM and LB to confirm transcription accuracy.

### 2.4. Coding and Analysis

A reflexive thematic analysis approach, utilising a phenomenological and essentialist–realist framework, was used to analyse interview transcripts on a semantic level [34]. Data analysis was supported by NVivo V.12 (QSR International, 2017). The analysis involved multiple steps. Initially, three study authors (SC, PM, and LB) familiarised themselves with the data by reading interview transcripts and listening to interview recordings. Each author then independently analysed the interviews by generating initial codes for each line of data. Codes were subsequently reviewed and grouped into themes and subthemes. All interviews were double coded with SC coding 100% and PM and LB each coding 50% of the interviews. Codes and themes were discussed at frequent meetings to ensure that data were being interpreted consistently. Finally, PM and LB extracted quotes for each of the themes and subthemes. The final interview extracts that appear in this manuscript were chosen by SC and were chosen as they presented the clearest description of the themes/subthemes. The quotes were edited to the extent that filler words (e.g., “um” or “like”) and identifying information were removed and irrelevant sentences were replaced with ellipses (…).

## 3. Results

**Figure 1:**
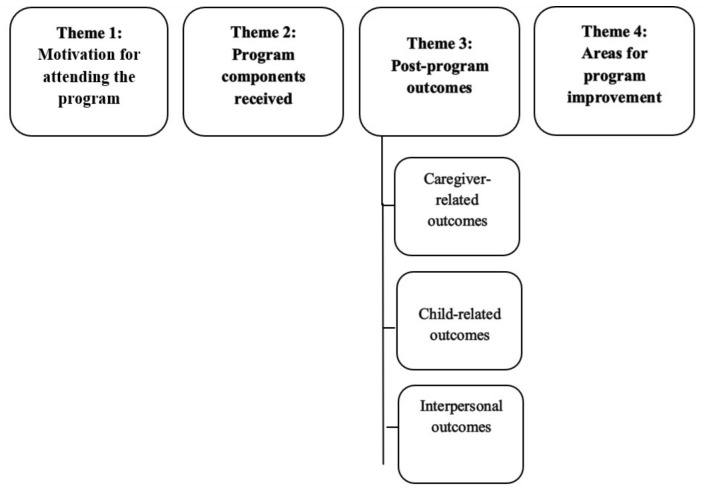
Themes and subthemes.

Four themes and three subthemes emerged from the data: (1) motivation for attending the program; (2) program components received; (3) post-program outcomes (subthemes: caregiver-related outcomes, child-related outcomes, and interpersonal outcomes); and (4) areas for program improvement. An overview of the themes and subthemes is provided in Figure 1.

### 3.1. Theme 1: Motivation for Attending the Program

Though not asked directly, most caregivers indicated who referred them to the program. Several services/service providers were mentioned as referral sources, including acute inpatient mental health services (*n* = 1), a mother and baby unit (*n* = 1), the Department of Communities and Justice (*n* = 1), a midwife (*n* = 1), Child and Family Health Nurses (*n* = 2), case workers/social workers (*n* = 2), and a Perinatal Infant Mental Health Service (*n* = 1). Two participants did not mention where they were referred from. One participant noted being transferred to Nurturing Connections by her clinician, who had taken up a job with the Nurturing Connections Program. When asked about their reasons for attending the services, caregivers indicated parenting support (Caregivers 1, 3, and 4), support in bonding with their child (Caregivers 6 and 11), and help with their mental health (Caregivers 2, 5, and 10).

“My child and family health nurse… recommended it to me because I’d struggled with postnatal depression and anxiety, and felt a disconnect from my daughter.”

(Caregiver 10, 13-month-old child)

“I knew he was mine, but it felt like I’d just been handed this baby and didn’t know what to do. It was hard to give him an identity beyond just “a baby,” and even harder to feel he was my baby. That disconnect made me feel guilty. I’d wanted to be a mum so badly, but when it happened, I didn’t feel the love I expected.”

(Caregiver 11, 13-month-old child)

One caregiver indicated that although they felt their relationship with their child was “not bad”, they themselves were “so exhausted” (Caregiver 9). Others commented that by attending the service they were hoping to “meet more people” (Caregiver 12) and feel less “alone” (Caregiver 2).

### 3.2. Theme 2: Program Components Received

All of the participants spoke of the wide range of support that they had received from the Nurturing Connections Program. The supports mentioned included meetings with the social worker, the psychiatrist, and/or the child and family health nurse; receiving home visits; receiving financial and practical support; and participating in group work. Most caregivers acknowledged receiving more than one type of support and noted the positive benefits associated with this, highlighting how beneficial access to a multidisciplinary team was for addressing the various needs often experienced by families with high vulnerability.

“I mainly engaged with [clinical], but I also met [the child and family health nurse] which was helpful early on when I had questions about my son. I also met the support worker, who helped me with my Centrelink application, [son’s] Medicare, and his birth certificate. That took a huge weight off me. I also met [psychiatrist] a couple of times.”

(Caregiver 7, 7-month-old child)

One caregiver described the support as feeling like “… I had an “umbrella”- someone to reach out to if I needed help, which I didn’t have before.” (Caregiver 11). Participants also spoke positively about their perceptions of the Nurturing Connections team members and the service as a whole. Team members were described as “lovely” (Caregiver 2), “beautiful” (Caregivers 1 and 2), “amazing” (Caregiver 6), and “open-minded” (Caregiver 12), while the program itself was viewed by most as “helpful” (Caregivers 4 and 5) and going “way above what [was] expected” (Caregiver 8).

“We were absolutely thrilled with all the support that we were given. Everyone’s been lovely. I really hope other families get the opportunity to have of this sort of supported service… I think it is first class.”.

(Caregiver 8, 2-year-old child)

### 3.3. Theme 3: Post Program Outcomes

#### 3.3.1. Caregiver-Related Outcomes

Most caregivers reported improvements in their own emotional regulation, mental health, and/or parenting self-efficacy because of the service. They also reported gaining new insight into their own child’s development.

“…I still get very upset with the kids, even though that’s not their fault. … we’re in the middle of, kind of working on that now… it’s something that’s getting better.”.

(Caregiver 1, 2-year-old child)

“Being part of Nurturing Connections had a good impact on my mental health. It’s made me more aware of things and helped me find ways to cope better.”.

(Caregiver 3, 4-month-old child)

Several participants noted attending a group while participating in the Nurturing Connections Program. The participants did not specify which group program they participated in; therefore, the feedback reflects their overall experience of group program participation. The majority of caregivers acknowledged learning new information during group sessions; their experience in meeting other caregivers who had similar parenting or life stress was, however, a recurring positive point of discussion.

“Meeting the other mums was lovely. It helped me see I wasn’t alone—that many of us had difficult journeys.”.

(Caregiver 10, 13-month-old child)

Two caregivers who participated in a group program found them less beneficial, particularly when they had multiple children or young babies.

“[Clinician] suggested courses, but I found them impractical with a young baby.”.

(Caregiver 6, 3.5-year-old child)

“I went once to their group, but [child] was too young compared to the other kids. It was hard to get there in the mornings.”.

(Caregiver 7, 7-month-old child)

#### 3.3.2. Child-Related Outcomes

In addition to reporting positive gains related to their parenting ability and mental and emotional health, most caregivers also commented on how the program benefited their children. The participants discussed their children’s health and/or developmental gains from one-on-one support provided by the Nurturing Connections team members or from receiving specialist services via referrals to other services. Some parents felt that because their own emotional wellbeing improved through the care they received, their children also benefited.

“He benefits because the ladies talk to him and give me good input. The child health nurse gives me advice on feeding and breastfeeding.”.

(Caregiver 3, 4-month-old child)

“My daughter has health concerns. A paediatric psychiatrist in the programme helped link us with specialists and testing, which was a big help.”.

(Caregiver 5, 2.5-year-old child)

#### 3.3.3. Interpersonal Outcomes

Many of the caregivers felt their relationship with their child had improved since participating in the program. The caregivers attributed these improvements to greater insights into child development, spending more quality time together, and greater parental capacity.

“… Now I see my son in a totally different, very positive light. I’m excited to spend time with him. The older he gets, the more I’ve realised I enjoy play where he gives something back—something I learned through Nurturing Connections.”

(Caregiver 11, 13-month-old child)

“They took short videos of me with my daughter… changing her nappy or playing. They later analyzed them and showed me snippets of great parenting moments. It was lovely because as a mum I’m rarely in the picture. Something mundane like changing a nappy turned out to be a positive bonding moment.”.

(Caregiver 5, 2.5-year-old child)

Some of the participants discussed how the program positively affected their relationships with their partners and their own parents. Relationships with partners were reported to have improved due to the implementation of a unified parenting method, that is, consistency between caregivers’ approach to raising their child, including boundaries, communication and expectations. Conversely, relationships with their own parents were often viewed differently when caregivers were encouraged to reflect on their own childhood.

“It helped guide my partner and me—gave us a way to work together, focus as a team, and improved our relationship with each other and with my daughter.”

(Caregiver 4, 4.5-year-old child)

“It’s brought up a lot of childhood feelings and helped me understand why I react the way I do. Motherhood makes you want to create a better life for your child, and that brings up trauma. I’ve been trying to face that trauma and it’s improved my relationship with my parents. Improving my relationship with my parents helps me have a better one with my son.”

(Caregiver 11, 13-month-old child)

Some of the participants reported not observing any changes in their relationship with their children or others.

“Participant: We had a good relationship, but I struggled with boundaries. I worried about setting limits, fearing it would ruin our bond because of my own relationship with my mum. As he got older and more defiant, I found it hard to take a leadership role without feeling cruel. During pregnancy I was also sick and distant from him, which made it harder.

Interviewer: Did the program help improve that relationship?

Participant: Not really… but I don’t think I was in the service long enough.”

(Caregiver 6, 3.5-year-old child).

“Interviewer: And your relationship with your partner—any impact?

Participant: Not really. He just goes on with things. He didn’t participate in the program—he was at work, though he came to some psychiatrist appointments.”

(Caregiver 5, 2.5-year-old child).

### 3.4. Theme 4: Areas for Program Improvement

The program was perceived positively by most of the participants, and when asked to reflect on ways the program could be improved, the common response referred to receiving clearer communication about the program. In particular, some saw a need for greater clarification regarding what the program involved and staff roles.

“I thought it would give me access to a lot of different providers—continuing midwife visits, a social worker, [occupational therapist], maybe even a psychologist. I expected to be assessed and then linked into those supports, especially around mental health.”.

(Caregiver 10, 13-month-old child)

“So when I was referred on, I expected Nurturing Connections would provide counseling too. Instead, I saw [clinician] weekly without really understanding her role. She was kind, but not a counselor… I think the service needs to better explain what staff roles are and how they’ll actually help you.”.

(Caregiver 6, 3.5-year-old child)

One participant expressed concern about having the child in the room when discussing certain topics (“I worried sometimes about her being in the room when we discussed her…” (Caregiver 4). While another indicated that “more balance” (Caregiver 10) in regard to involving the child in treatment was needed. Finally, two caregivers expressed worry about the potential “lack of support” (Caregiver 5) after being discharged from the program; however, it was acknowledged that service providers had reassured them that they could reengage with the service if required.

“It was a little bit scary when everybody went, but on the other hand [clinician] said, look, if you need to call us, call us. Don’t hesitate to call…”.

(Caregiver 8, 2-year-old child)

## 4. Discussion

This study was the first to evaluate caregivers’ perceptions of and experiences with the Nurturing Connections Program—a new Australian public mental health service for caregivers of young children mental health difficulties and complex needs. The four major themes that emerged from the data focused on reasons for accessing the program, experiences with the care received, perceptions of the program’s impact, and ways to improve the program.

The findings showed that Australian caregivers of young children, who experienced moderate-to-severe mental illness and complex needs, perceived the program as beneficial to themselves, their children, and/or their family relationships. The participants reported that receiving multidisciplinary care from experienced and caring staff was instrumental in reducing their distress and improving their own emotional and mental wellbeing, while attending groups helped them connect with others and reduce feelings of isolation. They also highlighted their improved understanding of child development and the closer relationships with their children following program completion. Furthermore, caregivers recognised the direct and indirect benefits their children were receiving because of their participation in the program. These findings are consistent with the literature showing that service users benefit when their multifaceted needs are addressed through the provision of comprehensive care [4,35]. Furthermore, these findings add to the evidence base showing that multidisciplinary team interventions focusing on caregiver mental health and psychosocial factors, child development, and the dyadic relationship lead to improved caregiver, child, and family outcomes in families with caregivers experiencing moderate-to-severe mental illness and complex needs [17,36].

Though all caregivers reported benefiting from the program, for some, this was not without its challenges. A couple of the participants noted that accessing groups with young children was unfeasible. This finding is in line with previous research showing that common logistic barriers, such as time and resources, inconvenient session times, and childcare, are factors that impact caregivers’ group attendance [17,37]. Patient-centred approaches and flexible program delivery are factors that have been highlighted as increasing caregiver group engagement [17,37].

Some of the participants also expressed confusion regarding program components or staff roles. As Nurturing Connections is based on a tiered model of care, participants’ needs are assessed preintervention and the supports/interventions provided are tailored on a needs basis [24]. Thus, in line with a tiered model of care, participants often do not receive all supports offered by the program. Early discussions regarding the program structure, however, appear not to have emphasised the tiered model of care, leaving some caregivers expecting access to all supports/interventions and feeling confused when these were not received.

In regard to uncertainty pertaining to staff roles, mental health services in Australia often hire staff members with a variety of allied health backgrounds, including psychology, occupational therapy, and social work, to provide counselling services. These allied health professionals have extensive knowledge of mental health and counselling; however, for service users who do not have a prior understanding of how mental health services operate, confusion can occur when, for example, they are assigned a staff member with an occupational therapy background to provide mental health interventions. Misunderstandings regarding service provision and the role that service providers play are not a unique experience for caregivers accessing the Nurturing Connections Program [38,39]. For example, in a longitudinal qualitative study exploring the views of peer-workers in Hong Kong, Tse et al. [39] found that service users often expressed uncertainty regarding the role of peer workers in the mental health system. Research has found that role confusion is not limited to service users but also impacts service providers working within the mental health system [40]. A qualitative study undertaken by Smith and Mackenzie [40], for example, showed that experienced nurses working in mental health settings expressed difficulty understanding the role that Occupational Therapists could play in these settings. Moving forward, detailed descriptions regarding how supports/interventions are provided by the service and the role of staff members would aid in reducing participant confusion and improving service engagement.

### Limitations and Future Research

This study was the first to evaluate caregivers’ perceptions of the Nurturing Connections Program, delivered to families across three LHDs in New South Wales, Australia. A limitation of this research was a lack of diversity in the sample, as all caregivers were female, and the majority were biological mothers. A further limitation was the unequal representation of participants from the metropolitan LHDs (75%) compared to the rural LHD (25%), especially as only one participant identified as Torres Strait Islander, restricting the generalisability of findings to rural areas. This is of particular importance given the higher percentage of Aboriginal and Torres Strait Islander families in rural and regional areas compared to metropolitan areas. Further research with a more representative sample, including fathers and kinship carers, would lead to a better understanding of the program’s acceptability and factors related to program engagement and retention. Finally, while this study provides insights into participants’ experiences with and perceptions of the program, it does not allow for conclusions regarding the program’s overall effectiveness in improving caregiver and child outcomes to be made. Future research would benefit from utilising systematic evaluation procedures, such as clinical trials, to determine whether the program results in quantifiable improvements in outcomes.

## 5. Conclusions

In conclusion, this study’s findings highlight caregivers’ experiences with and perceptions of the Nurturing Connections Program—a highly specialised Australian public mental health service targeted at caregivers with young children, moderate-to-severe mental illness and complex needs. While confusion regarding what the service offered and by whom was a challenge experienced by some, all of the caregivers reported positive outcomes in at least one area—for themselves, their children, and/or their interpersonal relationships. These findings provide support for the Nurturing Connections Program as an acceptable and beneficial relationship-focused intervention aligned with contemporary evidence that caregiver mental health treatment must extend beyond symptom reduction alone. Future research would benefit from utilising a more diverse and representative sample to further examine the program’s impact across different populations and contexts.

## Figures and Tables

**Table 1:** Nurturing Connections Program eligibility.

Eligibility Criteria	
1	Pregnant (>24 weeks)/primary caregiver of young children (conception to school age) with whom they reside.
2	Caregiver has a diagnosed moderate-to-severe and/or complex mental health disorder (based on referral or triage assessment).
3	The child–caregiver attachment relationship is negatively impacted by the caregiver’s mental health.
4	Caregiver experiences ≥ 2 psychosocial vulnerabilities, including economic hardship, domestic and family violence, limited social support, child protection history, and/or being a teenage parent.
5	Caregiver resides within the catchment area.
**Ineligibility Criteria**	
1	The caregiver is in an acute phase of their mental health disorder, which requires active care from acute mental health services.
2	The caregiver is engaged with alternative family focused programs (e.g., Whole Family Teams [31]).
3	The caregiver is experiencing active substance dependence or problematic substance use and is not willing to engage in treatment.
4	The caregiver is under a court-ordered parenting assessment or program (e.g., from the Children’s Court).

## Data Availability

Data available on request due to privacy/ethical restrictions.
